# Supplementary material for: Effects of Virtual Reality Interventions for Needle-Related Procedures in Patients with Cancer: A Systematic Review and Meta-Analysis
Source: Cancers (Basel). 2025 Jun 12;17(12):1954. doi: 10.3390/cancers17121954 (PMC12191400; doi:10.3390/cancers17121954)
Supplement: Supplementary file 1 [file cancers-17-01954-s001.zip › cancers-3677876-supplementary.pdf]

Table S1. PRISMA 2020 Checklist

Section and Topic	Item #	Checklist item	Location where item is reported
**TITLE**			
Title	1	Identify the report as a systematic review.	1
**ABSTRACT**			
Abstract	2	See the PRISMA 2020 for Abstracts checklist.	1
**INTRODUCTION**			
Rationale	3	Describe the rationale for the review in the context of existing knowledge.	2
Objectives	4	Provide an explicit statement of the objective(s) or question(s) the review addresses.	3
**METHODS**			
Eligibility criteria	5	Specify the inclusion and exclusion criteria for the review and how studies were grouped for the syntheses.	4
Information sources	6	Specify all databases, registers, websites, organisations, reference lists and other sources searched or consulted to identify studies. Specify the date when each source was last searched or consulted.	4
Search strategy	7	Present the full search strategies for all databases, registers and websites, including any filters and limits used.	4
Selection process	8	Specify the methods used to decide whether a study met the inclusion criteria of the review, including how many reviewers screened each record and each report retrieved, whether they worked independently, and if applicable, details of automation tools used in the process.	4
Data collection process	9	Specify the methods used to collect data from reports, including how many reviewers collected data from each report, whether they worked independently, any processes for obtaining or confirming data from study investigators, and if applicable, details of automation tools used in the process.	4
Data items	10a	List and define all outcomes for which data were sought. Specify whether all results that were compatible with each outcome domain in each study were sought (e.g. for all measures, time points, analyses), and if not, the methods used to decide which results to collect.	4
	10b	List and define all other variables for which data were sought (e.g. participant and intervention characteristics, funding sources). Describe any assumptions made about any missing or unclear information.	4
Study risk of bias assessment	11	Specify the methods used to assess risk of bias in the included studies, including details of the tool(s) used, how many reviewers assessed each study and whether they worked independently, and if applicable, details of automation tools used in the process.	4
Effect measures	12	Specify for each outcome the effect measure(s) (e.g. risk ratio, mean difference) used in the synthesis or presentation of results.	5
Synthesis methods	13a	Describe the processes used to decide which studies were eligible for each synthesis (e.g. tabulating the study intervention characteristics and comparing against the planned groups for each synthesis (item #5)).	5
	13b	Describe any methods required to prepare the data for presentation or synthesis, such as handling of missing summary statistics, or data conversions.	5

Section and Topic	Item #	Checklist item	Location where item is reported
	13c	Describe any methods used to tabulate or visually display results of individual studies and syntheses.	5
	13d	Describe any methods used to synthesize results and provide a rationale for the choice(s). If meta-analysis was performed, describe the model(s), method(s) to identify the presence and extent of statistical heterogeneity, and software package(s) used.	5
	13e	Describe any methods used to explore possible causes of heterogeneity among study results (e.g. subgroup analysis, meta-regression).	5
	13f	Describe any sensitivity analyses conducted to assess robustness of the synthesized results.	NA
Reporting bias assessment	14	Describe any methods used to assess risk of bias due to missing results in a synthesis (arising from reporting biases).	5
Certainty assessment	15	Describe any methods used to assess certainty (or confidence) in the body of evidence for an outcome.	5
**RESULTS**			
Study selection	16a	Describe the results of the search and selection process, from the number of records identified in the search to the number of studies included in the review, ideally using a flow diagram.	7
	16b	Cite studies that might appear to meet the inclusion criteria, but which were excluded, and explain why they were excluded.	7
Study characteristics	17	Cite each included study and present its characteristics.	8-14
Risk of bias in studies	18	Present assessments of risk of bias for each included study.	15
Results of individual studies	19	For all outcomes, present, for each study: (a) summary statistics for each group (where appropriate) and (b) an effect estimate and its precision (e.g. confidence/credible interval), ideally using structured tables or plots.	15
Results of syntheses	20a	For each synthesis, briefly summarise the characteristics and risk of bias among contributing studies.	6, 16
	20b	Present results of all statistical syntheses conducted. If meta-analysis was done, present for each the summary estimate and its precision (e.g. confidence/credible interval) and measures of statistical heterogeneity. If comparing groups, describe the direction of the effect.	15-16
	20c	Present results of all investigations of possible causes of heterogeneity among study results.	15-16
	20d	Present results of all sensitivity analyses conducted to assess the robustness of the synthesized results.	NA
Reporting biases	21	Present assessments of risk of bias due to missing results (arising from reporting biases) for each synthesis assessed.	15
Certainty of evidence	22	Present assessments of certainty (or confidence) in the body of evidence for each outcome assessed.	5, 47-49
**DISCUSSION**			
Discussion	23a	Provide a general interpretation of the results in the context of other evidence.	16-18

Section and Topic	Item #	Checklist item	Location where item is reported
	23b	Discuss any limitations of the evidence included in the review.	18
	23c	Discuss any limitations of the review processes used.	18
	23d	Discuss implications of the results for practice, policy, and future research.	18-19
**OTHER INFORMATION**			
Registration and protocol	24a	Provide registration information for the review, including register name and registration number, or state that the review was not registered.	3
	24b	Indicate where the review protocol can be accessed, or state that a protocol was not prepared.	3
	24c	Describe and explain any amendments to information provided at registration or in the protocol.	NA
Support	25	Describe sources of financial or non-financial support for the review, and the role of the funders or sponsors in the review.	19
Competing interests	26	Declare any competing interests of review authors.	19
Availability of data, code and other materials	27	Report which of the following are publicly available and where they can be found: template data collection forms; data extracted from included studies; data used for all analyses; analytic code; any other materials used in the review.	NA

From: Page MJ, McKenzie JE, Bossuyt PM, Boutron I, Hoffmann TC, Mulrow CD, et al. The PRISMA 2020 statement: an updated guideline for reporting systematic reviews. BMJ 2021;372:n71. doi: 10.1136/bmj.n71. This work is licensed under CC BY 4.0. To view a copy of this license, visit https://creativecommons.org/licenses/by/4.0/

Table S2. Detailed search strategies of 11 electronic databases

Databases	Search Strategy	Results
**CINAHL**	**#S1** (MH "Virtual Reality+") OR (MH "Virtual Reality Exposure Therapy") OR (MH "Smart Glasses")	7,469
	**#S2** TI (VR OR Virtual Realit* OR Virtual therap* OR Virtual environment OR Virtual treatment OR Smartglass* OR Head mounted display OR Head up display OR Head worn display OR Hmd OR Immersive OR Oculus OR Google glass* OR Goggles) OR AB (VR OR Virtual Realit* OR Virtual therap* OR Virtual environment OR Virtual treatment OR Smartglass* OR Head mounted display OR Head up display OR Head worn display OR Hmd OR Immersive OR Oculus OR Google glass* OR Goggles)	18,842
	**#S3** S1 OR S2	21,709
	**#S4** (MH "Neoplasms+")	97,237
	**#S5** TI (Benign neoplasm OR Cancer* OR Chemotherap* OR Malignan* OR Neoplasm OR Neoplasia* OR Oncology OR Tumor*) OR AB (Benign neoplasm OR Cancer* OR Chemotherap* OR Malignan* OR Neoplasm OR Neoplasia* OR Oncology OR Tumor*)	754,248
	**#S6** S4 OR S5	773,764
	**#S7** (MH "Vascular Access Devices+") OR (MH "Vascular Access Devices, Implantable") OR (MH "Biopsy, Needle") OR (MH "Needles") OR (MH "Paracentesis+") OR (MH "Spinal Puncture") OR (MH "Thoracentesis") OR (MH "Catheterization, Peripheral Central Venous") OR (MH "Catheterization+") OR (MH "Catheterization, Peripheral+") OR (MH "Wounds and Injuries+")	439,960
	**#S8** TI (Ablation OR Arterial line* OR Aspiration* OR Bone marrow biops* OR Blood sampl* OR Blood specimen collection OR Cannulation OR Catheter* OR Chemotherap* OR Central Venous Catheter OR CVC OR Dressing Drip infusion* OR Epidural* OR Extradural* OR Inject* OR Injur* Intra-Arterial line* OR Intraosseous infusion OR Intrathecal injection* OR	526,247

	Implantation*OR Invasive procedure* OR Needle* OR Needle localization OR Needle-related OR Needle decompression OR Nerve block OR Paracentesis OR Peripherally inserted central catheter line OR PICC OR Port OR Port-A-Cath OR Port A Cath OR Spinal puncture* OR Spinal tap* OR Suture* OR Thoracentesis OR Vaccin* OR Vascular Cathet* OR Vascular access port OR Venipuncture OR Venous port OR Venous access OR Venous reservoir* OR Venous cannulat* OR Vascular Access Port* OR Vascular catheter* OR Lumbar puncture* OR Lumbar aspirat* OR Painful procedure* OR Wound*) OR AB (Ablation OR Arterial line* OR Aspiration* OR Bone marrow biops* OR Blood sampl* OR Blood specimen collection OR Cannulation OR Catheter* OR Chemotherap* OR Central Venous Catheter OR CVC OR Dressing Drip infusion* OR Epidural* OR Extradural* OR Inject* OR Injur* Intra-Arterial line* OR Intraosseous infusion OR Intrathecal injection* OR Implantation*OR Invasive procedure* OR Needle* OR Needle localization OR Needle-related OR Needle decompression OR Nerve block OR Paracentesis OR Peripherally inserted central catheter line OR PICC OR Port OR Port-A-Cath OR Port A Cath OR Spinal puncture* OR Spinal tap* OR Suture* OR Thoracentesis OR Vaccin* OR Vascular Cathet* OR Vascular access port OR Venipuncture OR Venous port OR Venous access OR Venous reservoir* OR Venous cannulat* OR Vascular Access Port* OR Vascular catheter* OR Lumbar puncture* OR Lumbar aspirat* OR Painful procedure* OR Wound*)	
	**#S9** S7 OR S8	878,797
	**#S10** S3 AND S6 AND S9	287
**Cochrane**	**#1** MeSH descriptor: [Neoplasms] explode all trees	126,336
	**#2** (Benign neoplasm OR Cancer* OR Chemotherap* OR Malignan* OR Neoplasm OR Neoplasia* OR Oncology OR Tumor*):ti,ab,kw	297,606
	**#3** MeSH descriptor: [Virtual Reality] explode all trees	1,388

	**#4** MeSH descriptor: [Virtual Reality Exposure Therapy] explode all trees	371
	**#5** (VR OR Virtual Realit* OR Virtual therap* OR Virtual environment OR Virtual treatment OR Smartglass* OR Head mounted display OR Head up display OR Head worn display OR Hmd OR Immersive OR Oculus OR Google glass* OR Goggles):ti,ab,kw	14,171
	**#6** MeSH descriptor: [Catheters] explode all trees	2,827
	**#7** MeSH descriptor: [Vascular Access Devices] explode all trees	649
	**#8** MeSH descriptor: [Spinal Puncture] explode all trees	374
	**#9** MeSH descriptor: [Biopsy, Needle] explode all trees	1,574
	**#10** MeSH descriptor: [Thoracentesis] explode all trees	28
	**#11** MeSH descriptor: [Paracentesis] explode all trees	434
	**#12** MeSH descriptor: [Wounds and Injuries] explode all trees	39,636
	**#13** (Ablation OR Arterial line* OR Aspiration* OR Bone marrow biops* OR Blood sampl* OR Blood specimen collection OR Cannulation OR Catheter* OR Chemotherap* OR Central Venous Catheter OR CVC OR Dressing Drip infusion* OR Epidural* OR Extradural* OR Inject* OR Injur* Intra-Arterial line* OR Intraosseous infusion OR Intrathecal injection* OR Implantation*OR Invasive procedure* OR Needle* OR Needle localization OR Needle-related OR Needle decompression OR Nerve block OR Paracentesis OR Peripherally inserted central catheter line OR PICC OR Port OR Port-A-Cath OR Port A Cath OR Spinal puncture* OR Spinal tap* OR Suture* OR Thoracentesis OR Vaccin* OR Vascular Cathet* OR Vascular access port OR Venipuncture OR Venous port OR Venous access OR Venous reservoir* OR Venous cannulat* OR Vascular Access Port* OR Vascular catheter* OR Lumbar puncture* OR Lumbar aspirat* OR Painful procedure* OR Wound*):ti,ab,kw	500,245
	**#14** (#1 OR #2) AND (#3 OR #4 OR #5) AND (#6 OR #7 OR #8 OR #9 OR #10 OR #11 OR #12 OR #13)	700
**Embase**	**#1** 'virtual reality'/exp OR 'virtual reality	35,555

	exposure therapy'/exp OR 'virtual reality head mounted display'/exp OR 'smart glasses'/exp	
	**#2** vr:ti,ab,kw OR 'virtual realit*':ti,ab,kw OR 'virtual therap*':ti,ab,kw OR 'virtual environment':ti,ab,kw OR 'virtual treatment':ti,ab,kw OR smartglass*:ti,ab,kw OR 'head mounted display':ti,ab,kw OR 'head up display':ti,ab,kw OR 'head worn display':ti,ab,kw OR hmd:ti,ab,kw OR immersive:ti,ab,kw OR oculus:ti,ab,kw OR 'google glass*':ti,ab,kw OR goggles:ti,ab,kw	51,203
	**#3** #1 OR #2	63,278
	**#4** 'neoplasm'/exp	6,665,506
	**#5** 'benign neoplasm':ti,ab,kw OR cancer*:ti,ab,kw OR chemotherap*:ti,ab,kw OR malignan*:ti,ab,kw OR neoplasm:ti,ab,kw OR neoplasia*:ti,ab,kw OR oncology:ti,ab,kw OR tumor*:ti,ab,kw	5,847,909
	**#6** #4 OR #5	7,873,279
	**#7** 'vascular access device'/exp OR 'catheter'/exp OR 'thoracocentesis'/exp OR 'lumbar puncture'/exp OR 'paracentesis'/exp OR 'biopsy needle'/exp OR 'injury'/exp	3,419,752
	**#8** ablation:ti,ab,kw OR 'arterial line*':ti,ab,kw OR aspiration*:ti,ab,kw OR 'bone marrow biops*':ti,ab,kw OR 'blood sampl*':ti,ab,kw OR 'blood specimen collection':ti,ab,kw OR cannulation:ti,ab,kw OR catheter*:ti,ab,kw OR chemotherap*:ti,ab,kw OR 'central venous catheter':ti,ab,kw OR cvc:ti,ab,kw OR 'dressing drip infusion*':ti,ab,kw OR epidural*:ti,ab,kw OR extradural*:ti,ab,kw OR inject*:ti,ab,kw OR 'injur* intra-arterial line*':ti,ab,kw OR 'intraosseous infusion':ti,ab,kw OR 'intrathecal	4,279,081

	injection*':ti,ab,kw OR 'implantation*or invasive procedure*':ti,ab,kw OR needle*:ti,ab,kw OR 'needle localization':ti,ab,kw OR 'needle related':ti,ab,kw OR 'needle decompression':ti,ab,kw OR 'nerve block':ti,ab,kw OR paracentesis:ti,ab,kw OR 'peripherally inserted central catheter line':ti,ab,kw OR picc:ti,ab,kw OR port:ti,ab,kw OR 'port a cath':ti,ab,kw OR 'spinal puncture*':ti,ab,kw OR 'spinal tap*':ti,ab,kw OR suture*:ti,ab,kw OR thoracentesis:ti,ab,kw OR vaccin*:ti,ab,kw OR 'vascular cathet*':ti,ab,kw OR 'vascular access port':ti,ab,kw OR venipuncture:ti,ab,kw OR 'venous port':ti,ab,kw OR 'venous access':ti,ab,kw OR 'venous reservoir*':ti,ab,kw OR 'venous cannulat*':ti,ab,kw OR 'vascular access port*':ti,ab,kw OR 'vascular catheter*':ti,ab,kw OR 'lumbar puncture*':ti,ab,kw OR 'lumbar aspirat*':ti,ab,kw OR 'painful procedure*':ti,ab,kw OR wound*:ti,ab,kw	
	**#9** #7 OR #8	6,905,812
	**#10** #3 AND #6 AND #9	1,170
**IEEE Xplore**	("All Metadata":VR OR "All Metadata":Virtual Realit* OR "All Metadata":Virtual therapy OR "All Metadata":Virtual environment OR "All Metadata":Virtual treatment OR "All Metadata":Smartglass* OR "All Metadata":Head mounted display OR "All Metadata":Head up display OR "All Metadata":Head worn display OR "All Metadata":Hmd OR "All Metadata":Immersive OR "All Metadata":Oculus OR "All Metadata":Google glass* OR "All Metadata":Goggles) AND ("All Metadata":Benign neoplasm* OR "All Metadata":Cancer OR "All Metadata":Chemotherap* OR "All	156

	Metadata":Malignan* OR "All Metadata":Neoplasm OR "All Metadata":Oncology OR "All Metadata":Tumor) AND ("All Metadata":Ablation OR "All Metadata":Arterial line OR "All Metadata":Aspiration OR "All Metadata":Bone marrow biopsy OR "All Metadata":Blood sample OR "All Metadata":Blood specimen collection OR "All Metadata":Cannulation OR "All Metadata":Catheter OR "All Metadata":Central Venous Catheter OR "All Metadata":CVC OR "All Metadata":Dressing OR "All Metadata":Drip infusion OR "All Metadata":Epidural OR "All Metadata":Extradural OR "All Metadata":Inject* OR "All Metadata":Injur* Intra-Arterial line OR "All Metadata":Intraosseous infusion OR "All Metadata": Intrathecal injection OR "All Metadata":Implantation OR "All Metadata": Invasive procedure OR "All Metadata":Needle OR "All Metadata":Needle localization OR "All Metadata": Needle-related OR "All Metadata":Needle decompression OR "All Metadata":Nerve block OR "All Metadata":Paracentesis OR "All Metadata": Peripherally inserted central catheter line OR "All Metadata":PICC OR "All Metadata": Port OR "All Metadata":Port-A-Cath OR "All Metadata":Port A Cath OR "All Metadata":Spinal puncture OR "All Metadata":Spinal tap OR "All Metadata":Suture OR "All Metadata":Thoracentesis OR "All Metadata":Vaccin* OR "All Metadata":Vascular Catheter OR "All Metadata":Vascular access port OR "All Metadata":Venipuncture OR "All Metadata":Venous port OR "All Metadata":Venous access OR "All Metadata":Venous reservoir OR "All Metadata":Venous cannulation OR "All Metadata":Vascular Access Port OR "All Metadata":Vascular catheter OR "All Metadata":Lumbar puncture OR "All Metadata":Lumbar aspiration OR "All Metadata": Painful procedure OR "All Metadata":Wound)	

**MEDLINE**	**#1** exp Virtual Reality/ or exp Virtual Reality Exposure Therapy/ or exp Smart Glasses/	9,635
	**#2** (VR or Virtual Realit* or Virtual therap* or Virtual environment or Virtual treatment or Smartglass* or Head mounted display or Head up display or Head worn display or Hmd or Immersive or Oculus or Google glass* or Goggles).ab,ti,tw.	38,026
	**#3** #1 OR #2	39,482
	**#4** exp Neoplasms/	4,114,466
	**#5** (Benign neoplasm or Cancer* or Chemotherap* or Malignan* or Neoplasm or Neoplasia* or Oncology or Tumor*).ab,ti,tw.	4,650,636
	**#6** #4 OR #5	5,711,501
	**#7** exp Catheters/ or exp Central Venous Catheters/ or exp Thoracentesis/ or exp Paracentesis/ or exp Vascular Access Devices/ or exp Spinal Puncture/ or exp Biopsy, Needle/	131,815
	**#8** (Ablation or Arterial line* or Aspiration* or Bone marrow biops* or Blood sampl* or Blood specimen collection or Cannulation or Catheter* or Chemotherap* or Central Venous Catheter or CVC or Dressing Drip infusion* or Epidural* or Extradural* or Inject* or Injur* Intra-Arterial line* or Intraosseous infusion or Intrathecal injection* or Implantation*OR Invasive procedure* or Needle* or Needle localization or Needle-related or Needle decompression or Nerve block or Paracentesis or Peripherally inserted central catheter line or PICC or Port or Port-A-Cath or Port A Cath or Spinal puncture* or Spinal tap* or Suture* or Thoracentesis or Vaccin* or Vascular Cathet* or Vascular access port or Venipuncture or Venous port or Venous access or Venous reservoir* or Venous cannulat* or Vascular Access Port* or Vascular catheter* or Lumbar puncture* or Lumbar aspirat* or Painful procedure* or Wound*).ab,ti,tw.	2,952,210
	**#9** #7 OR #8	3,005,745
	**#10** #3 AND #6 AND #9	312

**ProQuest (Dissertations and These global)**	**S1** MAINSUBJECT.EXACT("Virtual reality") OR noft(VR OR Virtual Realit* OR Virtual therap* OR Virtual environment OR Virtual treatment OR Smartglass* OR Head mounted display OR Head up display OR Head worn display OR Hmd OR Immersive OR Oculus OR Google glass* OR Goggles)	30,133
	**S2** MAINSUBJECT.EXACT("Tumors") OR noft(Benign neoplasm OR Cancer* OR Chemotherap* OR Malignan* OR Neoplasm OR Neoplasia* OR Oncology OR Tumor*)	172,772
	**S3** MAINSUBJECT.EXACT("Venous access") OR MAINSUBJECT.EXACT("Ablation") OR MAINSUBJECT.EXACT("Biopsy") OR noft(Ablation OR Arterial line* OR Aspiration* OR Bone marrow biops* OR Blood sampl* OR Blood specimen collection OR Cannulation OR Catheter* OR Chemotherap* OR Central Venous Catheter OR CVC OR Dressing Drip infusion* OR Epidural* OR Extradural* OR Inject* OR Injur* Intra-Arterial line* OR Intraosseous infusion OR Intrathecal injection* OR Implantation*OR Invasive procedure* OR Needle* OR Needle localization OR Needle-related OR Needle decompression OR Nerve block OR Paracentesis OR Peripherally inserted central catheter line OR PICC OR Port OR Port-A-Cath OR Port A Cath OR Spinal puncture* OR Spinal tap* OR Suture* OR Thoracentesis OR Vaccin* OR Vascular Cathet* OR Vascular access port OR Venipuncture OR Venous port OR Venous access OR Venous reservoir* OR Venous cannulat* OR Vascular Access Port* OR Vascular catheter* OR Lumbar puncture* OR Lumbar aspirat* OR Painful procedure* OR Wound*)	222,073
	**S4** [S1] AND [S2] AND [S3]	133
**PsycINFO**	**#1** exp Virtual Reality/ or exp Virtual Reality Exposure Therapy/ or exp Smart Glasses/	1,4689
	**#2** (VR or Virtual Realit* or Virtual therap* or Virtual environment or Virtual treatment or Smartglass* or Head mounted display or Head up display or Head worn display or	18,405

	Hmd or Immersive or Oculus or Google glass* or Goggles).ab,ti,tw.	
	**#3** #1 OR #2	22,718
	**#4** exp Neoplasms/	68,343
	**#5** (Benign neoplasm or Cancer* or Chemotherap* or Malignan* or Neoplasm or Neoplasia* or Oncology or Tumor*).ab,ti,tw.	103,322
	**#6** #4 OR #5	106,684
	**#7** (Ablation or Arterial line* or Aspiration* or Bone marrow biops* or Blood sampl* or Blood specimen collection or Cannulation or Catheter* or Chemotherap* or Central Venous Catheter or CVC or Dressing Drip infusion* or Epidural* or Extradural* or Inject* or Injur* Intra-Arterial line* or Intraosseous infusion or Intrathecal injection* or Implantation*OR Invasive procedure* or Needle* or Needle localization or Needle-related or Needle decompression or Nerve block or Paracentesis or Peripherally inserted central catheter line or PICC or Port or Port-A-Cath or Port A Cath or Spinal puncture* or Spinal tap* or Suture* or Thoracentesis or Vaccin* or Vascular Cathet* or Vascular access port or Venipuncture or Venous port or Venous access or Venous reservoir* or Venous cannulat* or Vascular Access Port* or Vascular catheter* or Lumbar puncture* or Lumbar aspirat* or Painful procedure* or Wound*).ab,ti,tw.	136,862
	**#8** exp Catheter/ or exp Central Venous Catheters/ or exp Thoracentesis/ or exp Paracentesis/ or exp Vascular Access Devices/ or exp Spinal Puncture/ or exp Biopsy, Needle/	550
	**#9** #7 OR #8	136,894
	**#10** #3 AND #6 AND #9	55
**PubMed**	**#1** "Vascular Access Devices"[MeSH Terms] OR "biopsy, needle"[MeSH Terms] OR "Paracentesis"[MeSH Terms] OR "Spinal Puncture"[MeSH Terms] OR "Thoracentesis"[MeSH Terms] OR "Catheterization"[MeSH Terms] OR "Wounds and Injuries"[MeSH Terms]	1,356,901

	**#2** "Ablation"[Title/Abstract] OR "arterial line*"[Title/Abstract] OR "aspiration*"[Title/Abstract] OR "bone marrow biops*"[Title/Abstract] OR "blood sampl*"[Title/Abstract] OR "blood specimen collection"[Title/Abstract] OR "Cannulation"[Title/Abstract] OR "catheter*"[Title/Abstract] OR "chemotherap*"[Title/Abstract] OR "central venous catheter"[Title/Abstract] OR "CVC"[Title/Abstract] OR (("bandages"[MeSH Terms] OR "bandages"[All Fields] OR "dressing"[All Fields] OR "dressings"[All Fields] OR "dress"[All Fields] OR "dressed"[All Fields] OR "dresses"[All Fields] OR "dressing s"[All Fields]) AND "drip infusion*"[Title/Abstract]) OR "epidural*"[Title/Abstract] OR "extradural*"[Title/Abstract] OR "inject*"[Title/Abstract] OR ("injur*"[All Fields] AND "intra arterial line*"[Title/Abstract]) OR "intraosseous infusion"[Title/Abstract] OR "intrathecal injection*"[Title/Abstract] OR ("implantation*or"[All Fields] AND "invasive procedure*"[Title/Abstract]) OR "needle*"[Title/Abstract] OR "needle localization"[Title/Abstract] OR "Needle-related"[Title/Abstract] OR "needle decompression"[Title/Abstract] OR "nerve block"[Title/Abstract] OR "Paracentesis"[Title/Abstract] OR "peripherally inserted central catheter line"[Title/Abstract] OR "PICC"[Title/Abstract] OR "Port"[Title/Abstract] OR "Port-A-Cath"[Title/Abstract] OR "Port-A-Cath"[Title/Abstract] OR "spinal puncture*"[Title/Abstract] OR "spinal tap*"[Title/Abstract] OR "suture*"[Title/Abstract] OR "Thoracentesis"[Title/Abstract] OR "vaccin*"[Title/Abstract] OR "vascular cathet*"[Title/Abstract] OR "vascular access port"[Title/Abstract] OR "Venipuncture"[Title/Abstract] OR "venous port"[Title/Abstract] OR "venous access"[Title/Abstract] OR "venous reservoir*"[Title/Abstract] OR "venous cannulat*"[Title/Abstract] OR "vascular access	3,011,752

	port*"[Title/Abstract] OR "vascular catheter*"[Title/Abstract] OR "lumbar puncture*"[Title/Abstract] OR (("lumbarised"[All Fields] OR "lumbarization"[All Fields] OR "lumbarized"[All Fields] OR "lumbars"[All Fields] OR "lumbosacral region"[MeSH Terms] OR ("lumbosacral"[All Fields] AND "region"[All Fields]) OR "lumbosacral region"[All Fields] OR "Lumbar"[All Fields]) AND "aspirat*"[Title/Abstract]) OR "painful procedure*"[Title/Abstract] OR "wound*"[Title/Abstract]	
	**#3** "Vascular Access Devices"[MeSH Terms] OR "biopsy, needle"[MeSH Terms] OR "Paracentesis"[MeSH Terms] OR "Spinal Puncture"[MeSH Terms] OR "Thoracentesis"[MeSH Terms] OR "Catheterization"[MeSH Terms] OR "Wounds and Injuries"[MeSH Terms] OR ("Ablation"[Title/Abstract] OR "arterial line*"[Title/Abstract] OR "aspiration*"[Title/Abstract] OR "bone marrow biops*"[Title/Abstract] OR "blood sampl*"[Title/Abstract] OR "blood specimen collection"[Title/Abstract] OR "Cannulation"[Title/Abstract] OR "catheter*"[Title/Abstract] OR "chemotherap*"[Title/Abstract] OR "central venous catheter"[Title/Abstract] OR "CVC"[Title/Abstract] OR (("bandages"[MeSH Terms] OR "bandages"[All Fields] OR "dressing"[All Fields] OR "dressings"[All Fields] OR "dress"[All Fields] OR "dressed"[All Fields] OR "dresses"[All Fields] OR "dressing s"[All Fields]) AND "drip infusion*"[Title/Abstract]) OR "epidural*"[Title/Abstract] OR "extradural*"[Title/Abstract] OR "inject*"[Title/Abstract] OR ("injur*"[All Fields] AND "intra arterial line*"[Title/Abstract]) OR "intraosseous infusion"[Title/Abstract] OR "intrathecal injection*"[Title/Abstract] OR ("implantation*or"[All Fields] AND "invasive procedure*"[Title/Abstract]) OR "needle*"[Title/Abstract] OR "needle	4,079,250

	localization"[Title/Abstract] OR "Needle-related"[Title/Abstract] OR "needle decompression"[Title/Abstract] OR "nerve block"[Title/Abstract] OR "Paracentesis"[Title/Abstract] OR "peripherally inserted central catheter line"[Title/Abstract] OR "PICC"[Title/Abstract] OR "Port"[Title/Abstract] OR "Port-A-Cath"[Title/Abstract] OR "Port-A-Cath"[Title/Abstract] OR "spinal puncture*"[Title/Abstract] OR "spinal tap*"[Title/Abstract] OR "suture*"[Title/Abstract] OR "Thoracentesis"[Title/Abstract] OR "vaccin*"[Title/Abstract] OR "vascular cathet*"[Title/Abstract] OR "vascular access port"[Title/Abstract] OR "Venipuncture"[Title/Abstract] OR "venous port"[Title/Abstract] OR "venous access"[Title/Abstract] OR "venous reservoir*"[Title/Abstract] OR "venous cannulat*"[Title/Abstract] OR "vascular access port*"[Title/Abstract] OR "vascular catheter*"[Title/Abstract] OR "lumbar puncture*"[Title/Abstract] OR (("lumbarised"[All Fields] OR "lumbarization"[All Fields] OR "lumbarized"[All Fields] OR "lumbars"[All Fields] OR "lumbosacral region"[MeSH Terms] OR ("lumbosacral"[All Fields] AND "region"[All Fields]) OR "lumbosacral region"[All Fields] OR "Lumbar"[All Fields]) AND "aspirat*"[Title/Abstract]) OR "painful procedure*"[Title/Abstract] OR "wound*"[Title/Abstract])	
	**#4** "Neoplasms"[MeSH Terms]	4,106,918
	**#5** "benign neoplasm"[Title/Abstract] OR "cancer*"[Title/Abstract] OR "chemotherap*"[Title/Abstract] OR "malignan*"[Title/Abstract] OR "Neoplasm"[Title/Abstract] OR "neoplasia*"[Title/Abstract] OR "Oncology"[Title/Abstract] OR "tumor*"[Title/Abstract]	4,139,113

	**#6** "Neoplasms"[MeSH Terms] OR "benign neoplasm"[Title/Abstract] OR "cancer*"[Title/Abstract] OR "chemotherap*"[Title/Abstract] OR "malignan*"[Title/Abstract] OR "Neoplasm"[Title/Abstract] OR "neoplasia*"[Title/Abstract] OR "Oncology"[Title/Abstract] OR "tumor*"[Title/Abstract]	5,477,667
	**#7** "Virtual Reality"[MeSH Terms] OR "Virtual Reality Exposure Therapy"[MeSH Terms] OR "Smart Glasses"[MeSH Terms]	9,505
	**#8** "VR"[Title/Abstract] OR "virtual realit*"[Title/Abstract] OR "virtual therap*"[Title/Abstract] OR "virtual environment"[Title/Abstract] OR "virtual treatment"[Title/Abstract] OR "smartglass*"[Title/Abstract] OR "head mounted display"[Title/Abstract] OR "head up display"[Title/Abstract] OR "head worn display"[Title/Abstract] OR "Hmd"[Title/Abstract] OR "Immersive"[Title/Abstract] OR "Oculus"[Title/Abstract] OR "google glass*"[Title/Abstract] OR "Goggles"[Title/Abstract]	39,647
	**#9** "Virtual Reality"[MeSH Terms] OR "Virtual Reality Exposure Therapy"[MeSH Terms] OR "Smart Glasses"[MeSH Terms] OR "VR"[Title/Abstract] OR "virtual realit*"[Title/Abstract] OR "virtual therap*"[Title/Abstract] OR "virtual environment"[Title/Abstract] OR "virtual treatment"[Title/Abstract] OR "smartglass*"[Title/Abstract] OR "head mounted display"[Title/Abstract] OR "head up display"[Title/Abstract] OR "head worn display"[Title/Abstract] OR "Hmd"[Title/Abstract] OR "Immersive"[Title/Abstract] OR "Oculus"[Title/Abstract] OR "google glass*"[Title/Abstract] OR "Goggles"[Title/Abstract]	40,988
	**#10** ("Vascular Access Devices"[MeSH Terms] OR "biopsy, needle"[MeSH Terms] OR "Paracentesis"[MeSH Terms] OR	350

	"Spinal Puncture"[MeSH Terms] OR "Thoracentesis"[MeSH Terms] OR "Catheterization"[MeSH Terms] OR "Wounds and Injuries"[MeSH Terms] OR ("Ablation"[Title/Abstract] OR "arterial line*"[Title/Abstract] OR "aspiration*"[Title/Abstract] OR "bone marrow biops*"[Title/Abstract] OR "blood sampl*"[Title/Abstract] OR "blood specimen collection"[Title/Abstract] OR "Cannulation"[Title/Abstract] OR "catheter*"[Title/Abstract] OR "chemotherap*"[Title/Abstract] OR "central venous catheter"[Title/Abstract] OR "CVC"[Title/Abstract] OR (("bandages"[MeSH Terms] OR "bandages"[All Fields] OR "dressing"[All Fields] OR "dressings"[All Fields] OR "dress"[All Fields] OR "dressed"[All Fields] OR "dresses"[All Fields] OR "dressing s"[All Fields]) AND "drip infusion*"[Title/Abstract]) OR "epidural*"[Title/Abstract] OR "extradural*"[Title/Abstract] OR "inject*"[Title/Abstract] OR ("injur*"[All Fields] AND "intra arterial line*"[Title/Abstract]) OR "intraosseous infusion"[Title/Abstract] OR "intrathecal injection*"[Title/Abstract] OR ("implantation*or"[All Fields] AND "invasive procedure*"[Title/Abstract]) OR "needle*"[Title/Abstract] OR "needle localization"[Title/Abstract] OR "Needle-related"[Title/Abstract] OR "needle decompression"[Title/Abstract] OR "nerve block"[Title/Abstract] OR "Paracentesis"[Title/Abstract] OR "peripherally inserted central catheter line"[Title/Abstract] OR "PICC"[Title/Abstract] OR "Port"[Title/Abstract] OR "Port-A-Cath"[Title/Abstract] OR "Port-A-Cath"[Title/Abstract] OR "spinal puncture*"[Title/Abstract] OR "spinal tap*"[Title/Abstract] OR "suture*"[Title/Abstract] OR "Thoracentesis"[Title/Abstract] OR "vaccin*"[Title/Abstract] OR "vascular cathet*"[Title/Abstract] OR "vascular access port"[Title/Abstract] OR "Venipuncture"[Title/Abstract] OR "venous port"[Title/Abstract] OR "venous access"[Title/Abstract]	

	OR "venous reservoir*"[Title/Abstract] OR "venous cannulat*"[Title/Abstract] OR "vascular access port*"[Title/Abstract] OR "vascular catheter*"[Title/Abstract] OR "lumbar puncture*"[Title/Abstract] OR (("lumbarised"[All Fields] OR "lumbarization"[All Fields] OR "lumbarized"[All Fields] OR "lumbars"[All Fields] OR "lumbosacral region"[MeSH Terms] OR ("lumbosacral"[All Fields] AND "region"[All Fields]) OR "lumbosacral region"[All Fields] OR "Lumbar"[All Fields]) AND "aspirat*"[Title/Abstract]) OR "painful procedure*"[Title/Abstract] OR "wound*"[Title/Abstract])) AND ("Neoplasms"[MeSH Terms] OR ("benign neoplasm"[Title/Abstract] OR "cancer*"[Title/Abstract] OR "chemotherap*"[Title/Abstract] OR "malignan*"[Title/Abstract] OR "Neoplasm"[Title/Abstract] OR "neoplasia*"[Title/Abstract] OR "Oncology"[Title/Abstract] OR "tumor*"[Title/Abstract])) AND ("Virtual Reality"[MeSH Terms] OR "Virtual Reality Exposure Therapy"[MeSH Terms] OR "Smart Glasses"[MeSH Terms] OR ("VR"[Title/Abstract] OR "virtual realit*"[Title/Abstract] OR "virtual therap*"[Title/Abstract] OR "virtual environment"[Title/Abstract] OR "virtual treatment"[Title/Abstract] OR "smartglass*"[Title/Abstract] OR "head mounted display"[Title/Abstract] OR "head up display"[Title/Abstract] OR "head worn display"[Title/Abstract] OR "Hmd"[Title/Abstract] OR "Immersive"[Title/Abstract] OR "Oculus"[Title/Abstract] OR "google glass*"[Title/Abstract] OR "Goggles"[Title/Abstract]))	
**Scopus**	**1** TITLE-ABS-KEY ("virtual w/3 realit*" OR "virtual realit*" OR "virtual therap*" OR "virtual environment" OR "virtual treatment" OR smartglass* OR "head mounted display" OR "head up display" OR "head worn	269,731

	display" OR hmd OR immersive OR oculus OR "google glass*" OR goggles)	
	**2** TITLE-ABS-KEY (ablation OR "arterial line*" OR aspiration* OR "bone marrow biops*" OR "blood sampl*" OR "blood specimen collection" OR cannulat* OR catheter* OR chemotherap* OR " central venous catheter" OR cvc OR dressing OR " drip infusion*" OR epidural* OR extradural* OR inject* OR injur* "i ntra-arterial w/3 line*" OR "intraosseous infusion" OR "intrathecal injection*" OR implantation* OR " invasive procedure*" OR needle* OR "needle localization" OR "needle w/3 procedure*" OR "needle decompression" OR "nerve block" OR paracentesis OR "peripherally inserted central catheter" OR picc OR port OR port-a-cath OR "port a cath" OR "spinal puncture*" OR "spinal tap*" OR suture* OR thoracentesis OR vaccin* OR "vascular cathet*" OR "vascular w/3 port" OR venipuncture OR venous AND port OR "venous access" OR "venous reservoir*" OR "venous cannulat*" OR "vascular catheter*" OR "lumbar puncture*" OR "lumbar aspirat*" OR "painful procedure*" OR wound*)	67,402
	**3** TITLE-ABS-KEY ("benign neoplasm*" OR cancer* OR chemotherap* OR malignan* OR ne oplasm OR neoplasia* OR oncology OR tumor*)	6,881,374
	**4** (TITLE-ABS-KEY ("virtual w/3 realit*" OR "virtual realit*" OR "virtual therap*" OR "virtual environment" OR "virtual treatment" OR smartglass* OR "head mounted display" OR "head up display" OR "head worn display" OR hmd OR immersive OR oculus OR "google glass*" OR goggles) AND TITLE-ABS-KEY (ablation OR "arterial line*" OR aspiration* OR "bone	1,854

	marrow biops*" OR "blood sampl*" OR "blood specimen collection" OR cannulat* OR catheter* OR chemotherap* OR " central venous catheter" OR cvc OR dressing OR " drip infusion*" OR epidural* OR extradural* OR inject* OR injur* "i ntra-arterial w/3 line*" OR "intraosseous infusion" OR "intrathecal injection*" OR implantation* OR " invasive procedure*" OR needle* OR "needle localization" OR "needle w/3 procedure*" OR "needle decompression" OR "nerve block" OR paracentesis OR "peripherally inserted central catheter" OR picc OR port OR port-a-cath OR "port a cath" OR "spinal puncture*" OR "spinal tap*" OR suture* OR thoracentesis OR vaccin* OR "vascular cathet*" OR "vascular w/3 port" OR venipuncture OR venous AND port OR "venous access" OR "venous reservoir*" OR "venous cannulat*" OR "vascular catheter*" OR "lumbar puncture*" OR "lumbar aspirat*" OR "painful procedure*" OR wound*) AND TITLE-ABS-KEY ("benign neoplasm*" OR cancer* OR chemotherap* OR malignan* OR ne oplasm OR neoplasia* OR oncology OR tumor*))	
**Web of Science**	**#1** Benign neoplasm* OR Cancer* OR Chemotherap* OR Malignan* OR Neoplasm OR Neoplasia* OR Oncolog* OR Tumor* (Topic)	5,356,772
	**#2** TS=(Ablation OR Arterial line* OR Aspiration* OR Bone marrow biops* OR Blood sampl* OR Blood specimen collection OR Cannulation OR Catheter* OR Chemotherap* OR Central Venous Catheter OR CVC OR Dressing OR Drip infusion* OR Epidural* OR Extradural* OR Inject* OR Injur* Intra-Arterial line* OR Intraosseous infusion OR Intrathecal injection* OR Implantation*OR Invasive procedure* OR Needle* OR Needle localization OR Needle-related OR Needle decompression OR Nerve block OR Paracentesis OR Peripherally inserted central	4,14,693

	catheter line OR PICC OR Port OR Port-A-Cath OR Port A Cath OR Spinal puncture* OR Spinal tap* OR Suture* OR Thoracentesis OR Vaccin* OR Vascular Cathet* OR Vascular access port OR Venipuncture OR Venous port OR Venous access OR Venous reservoir* OR Venous cannulat* OR Vascular Access Port* OR Vascular catheter* OR Lumbar puncture* OR Lumbar aspirat* OR Painful procedure* OR Wound*)	
	**#3** VR OR Virtual Realit* OR Virtual therap* OR Virtual environment OR Virtual treatment OR Smartglass* OR Head mounted display OR Head up display OR Head worn display OR Hmd OR Immersive OR Oculus OR Google glass* OR Goggles (Topic)	236,266
	**#4** #1 AND #2 AND #3	1,845
**CNKI**	(Title, Keyword and Abstract: virtual reality (Fuzzy)) And (Title, Keyword and Abstract: cancer (Precise))	165

Table S3. Eligibility criteria

Criteria	Inclusion criteria	Exclusion criteria
Population	• Cancer patients receiving needle-related procedures regardless of age group or cancer type, including survivors, patients who are in active treatments	• Non-cancer patients<br>• Cancer patients receiving no needle-related procedures<br>• Healthy population
Intervention	Any type of VR-based intervention regardless of intervention setting, application, duration, number of sessions, etc.	Non-VR based intervention
Comparison	• Usual care<br>• Standard care	
Outcome	• Anxiety (primary)<br>• Pain (secondary)<br>• Depression (secondary)<br>• Fear (secondary)<br>• Pulse rate (secondary)<br>• Respiratory rate (secondary)	
Type of design	• Randomized controlled trials<br>• Mixed methods (RCT)	
Publication type	• Published research articles<br>• Unpublished thesis	
Language	• English<br>• Chinese	Languages other than English and Chinese

Table S4. GRADE Summary of Evidence Table

Certainty assessment							№ of patients		Effect		Certainty	Importance
**№ of studies**	**Study design**	**Risk of bias**	**Inconsistency**	**Indirectness**	**Imprecision**	**Other considerations**	**[Virtual reality]**	**[Usual care]**	**Relative (95% CI)**	**Absolute (95% CI)**		
**Anxiety**												
12	randomised trials	serious[a]	serious[b]	serious[c]	not serious[d]	none	476	473	-	SMD **1.74 SD lower** (2.47 lower to 1.01 lower)	⊕◯◯◯ Very low[a,b,c,d]	IMPORTANT
**Pain**												
8	randomised trials	serious[a]	serious[b]	serious[c]	not serious[d]	none	315	323	-	SMD **1.3 SD lower** (1.93 lower to 0.67 lower)	⊕◯◯◯ Very low[a,b,c,d]	IMPORTANT
**Depression**												
3	randomised trials	serious[a]	serious[e]	serious[c]	serious[f]	none	161	152	-	SMD **0.73 SD lower** (0.96 lower to 0.5 lower)	⊕◯◯◯ Very low[a,c,e,f]	IMPORTANT
**Fear**												

Certainty assessment							№ of patients		Effect		Certainty	Importance
**№ of studies**	**Study design**	**Risk of bias**	**Inconsistency**	**Indirectness**	**Imprecision**	**Other considerations**	**[Virtual reality]**	**[Usual care]**	**Relative (95% CI)**	**Absolute (95% CI)**		
3	randomised trials	serious[a]	not serious[g]	serious[c]	serious[h]	none	86	87	-	MD **1.31 lower** (1.56 lower to 1.06 lower)	⊕◯◯◯ Very low[a,c,g,h]	IMPORTANT
**Pulse rate**												
3	randomised trials	serious[a]	serious[b]	serious[c]	serious[h]	none	132	134	-	MD **0.25 higher** (14.32 lower to 14.81 higher)	⊕◯◯◯ Very low[a,b,c,h]	IMPORTANT
**Respiratory rate**												
2	randomised trials	serious[a]	serious[b]	serious[c]	serious[i]	none	100	101	-	MD **3.85 lower** (6.18 lower to 1.52 lower)	⊕◯◯◯ Very low[a,b,c,i]	IMPORTANT

CI: confidence interval; **MD:** mean difference; **SMD:** standardised mean difference

Explanations

a. All studies have performance bias

b. I square statistic 75%-100%, considerable heterogeneity

c. Participants from different countries, undergoing various format and duration of intervention

d. Total number of participants more than 400

e. I square statistic 30%-60%, moderate heterogeneity

f. Total number of participants less than 400

g. I square statistic <40%, low heterogeneity

h. Total number of participants less than 200

i. Total number of participants less than 300

Table S5 List of abbreviations

Abbreviation	Full Term
CI	Confidence Interval
CNKI	China National Knowledge Infrastructure
CINAHL	Cumulative Index to Nursing and Allied Health Literature
GRADE	Grading of Recommendations Assessments, Development, and Evaluation
HMD	Head Mounted Display
IEEE Xplore	Institute of Electrical and Electronics Engineers Xplore
MD	Mean Difference
NA	Not Applicable
NRP	Needle-Related Procedure
PICO	Population, Intervention, Comparison, Outcome
PRISMA	Preferred Reporting Items for Systematic Reviews and Meta-analysis
QOL	Quality Of Life
ROB	Risk Of Bias
RCT	Randomized Controlled Trial
RevMan	Review Manager
SD	Standard Deviations
SMD	Standard Mean Difference
SR	Systematic Review
VR	Virtual Reality
WHO	World Health Organization
